# The Association Between 7-To-10-year-old Children’s Leisure-time Physical Activities and Their Motor Behavior in a Virtual Playground Environment

**DOI:** 10.1177/00315125251347987

**Published:** 2025-05-30

**Authors:** Lise Storli, Håvard Lorås

**Affiliations:** 1 Department of Physical Education and Health, 208420 Queen Maud University College of Early Childhood Education, Trondheim, Norway; 2 Department of Teacher Education, Faculty of Social and Educational Sciences, 8018 NTNU, Trondheim, Norway

**Keywords:** motor development, movement variability, exercise, degrees of freedom, constraints

## Abstract

**Background:** Previous studies have shown mixed results for the relationship between physical activity experiences and gross motor development. **Purpose:** The aim of this study was to investigate the potential association between children’s level of engagement in selected leisure-time physical activities (active transport, sports, and play) and their emergent gross motor behavior measured in a virtual-reality playground environment. **Study Sample:** To this end, 247 children aged 7–10 years old (girls: *n* = 127; boys: *n* = 120) participated. **Reserach Design & Data Collection:** Children were provided with a three-minute playground scenario in virtual reality, developed for free exploration, play, and with the possibility to move around without specific instructions. An inertial motion capture system was used to assess motor behavior in the playground, and the children’s levels of engagement in weekly leisure-time physical activities were obtained through a parental questionnaire. **Results:** Correlation and principal component analysis were used to investigate the joint movement variability in the upper and lower body, while t-tests were applied to examine the potential differences in playground motor behavior and engagement in leisure-time physical activities. In statistical comparisons of children with high or low weekly activity rates, children with the highest levels of weekly engagement in play and active transport were found to have significantly higher upper-body movement variability. However, no other significant differences were found between high and low levels of leisure-time physical activity in terms of emergent motor behavior in the virtual reality playground. **Conclusion:** These findings emphasize the value of considering multiple dimensions of children’s leisure-time physical activities when studying its relation to the development of motor control and coordination in middle childhood.

## Introduction

A rich variety of physical experiences such as sports participation, playing outdoors in various terrains, or bicycling to school and friends is generally considered beneficial for child development, related to physical health, mental and emotional health, and cognitive development, as well as for specific skills that evolve throughout an individual’s life (Felfe et al., 2016; Haywood & Getchell, 2021; Logan et al., 2019; Overton, 2010). One benefit of such experiences is the continuous and dynamic development of *gross motor competence*, defined as the capability to perform a variety of movements involving several large muscle groups in order to engage in activities of daily living and physically demanding activities across the lifespan (Barnett et al., 2016; Gallahue & Ozmun, 2006). The concept includes the variation in children’s performance of fundamental motor skills with task-appropriate coordination and quality (Robinson et al., 2015), and it is typically operationalized through the interrelated categories of locomotion (e.g., skipping, running, and jumping), controlling objects (e.g., throwing and catching), and basic stability/balance. Gross motor competence is considered an essential part of healthy child development, and empirical findings support its association with various biopsychosocial domains, especially health-related fitness, participation in physical activities, prevention of obesity, and the lifelong maintenance of an active lifestyle (Barnett et al., 2022; D'Hondt et al., 2014; Lubans et al., 2010; Niemistö et al., 2020).

Physical activity, in principle, is any musculoskeletal movements leading to an increase in energy consumption beyond the basal metabolic rate (Caspersen et al., 1985), and its association with varying levels of gross motor competence in children has been systematically examined in the past two decades (Burton et al., 2023; Logan et al., 2015). These research efforts have been sparked by a conceptual model developed by Stodden et al. (2008) that aims to explain the role of motor competence in multiple health-related aspects of child development, including children’s physical activity. At the heart of the model’s framework is a reciprocal and developmentally dynamic direct relationship between motor competence and physical activity, the direction of which is mediated by development. This relationship suggests that children with higher motor competence are more likely to stay engaged in physical activities, which in turn further develops their motor competence (den Uil et al., 2023). Additionally, the model addresses the relationship between motor competence and developmental benefits, indicating that children who perceive themselves as more competent experience positive developmental outcomes. Lastly, health-related fitness is associated with maintaining a healthier weight status. If these three key relationships strengthen over time, they will lead to long-term positive experiences in physical activity, fitness, and overall health (Lima et al., 2022; Robinson et al., 2015).

According to the model, engaging in physical activity drives the level of motor competence in early childhood, as increased physical activity levels provide rich opportunities to stimulate the developmental processes associated with gross motor competence. However, in middle and late childhood, the relationship is reversed: higher gross motor competence offers a greater motor repertoire with which to participate in various physical activities, leading children with greater motor competence to self-select higher levels of physical activity and children with lower levels of gross motor competence to engage in lower levels of physical activity. Thus, in these developmental periods, the conceptual model considers gross motor competence as impacting physical activity levels. Studies have found significant differences in physical activity levels between children with lower motor competence and those with higher motor competence (Cantell & Crawford, 2008; Lopes et al., 2012); however, the model has faced criticism over its lack of clear evidence for the motor competence-to-physical activity pathway. The link between motor competence and physical activity is crucial for the interpretation of the relationship, but the only clear evidence supporting the association addresses the pathway from composite motor competence to physical activity, which demonstrates a positive relationship. It has been suggested that the significance of age in the motor competence-physical activity relationship needs to be further highlighted (Lima et al., 2022). However, a study found that the relationship between motor competence and physical activity strengthens with age (den Uil et al., 2023). For instance, children may not have a strong link between the two factors as their activities are more play-based for younger children; although, when entering middle childhood, those with higher motor competence are more likely to engage in physical activities, which further enhances motor skills (den Uil et al., 2023).

Methodological issues have been raised related to the examination of a potential relationship between children’s gross motor competence and physical activity levels in middle childhood. Physical activity is typically operationalized via accelerometers positioned at the waist and is expressed in metrics referring to overall intensity and/or duration (e.g., time spent in moderate-to-vigorous physical activity). Although shown to be a physiologically valid measure of exercise intensity in children (Dobell et al., 2019; Ekblom et al., 2012), this placement and use of accelerometers does not necessarily capture time spent conducting *varied* movement activities, which might have greater importance for gross motor competence (Pesce et al., 2016) irrespective of the intensity of the physical activity. This is especially important from an ecological perspective, as the movement experiences that emerge from being physically active at different intensities are dependent on the interaction between environmental, individual, and task constraints (Newell, 1986; Renshaw & Chow, 2019). Additionally, different types of physical activities might be conducted at the same physiological intensity but yield a substantial difference in terms of motor, perceptual, cognitive, and affective features (Chen & Sun, 2017; Grendstad & Hallén, 2024).

Leisure-time physical activities (LTPA) invite multiple intensity levels and movement experiences and typically refer to activities such as exercise and socializing during free time (Pressman et al., 2009). These activities include both organized (i.e., directed by adults) and non-organized (i.e., self-directed by children) physical activities. As such, this concept represents a shift in focus from the overall intensity of physical activity to the frequency of various *types* of physical activities. A well-studied component of LTPA is participation in sports. In middle childhood, improved motor-skill development provides more opportunities to engage in sports and other leisure activities (Stodden & Goodway, 2007). Studying the connection between motor competence and sports, Ulrich (1987) found that motor competence was significantly linked to participation in organized sports activities. Another study examined children between the ages of 6 and 10 years and found that children who performed better on physical fitness tests were more likely to participate in sports than those who performed worse on the test (Fransen et al., 2014). One study highlighted the significance of children’s involvement in organized physical activities to fundamental motor skill development, but that study also included organized physical activity during school time (Hardy et al., 2014). In contrast to these studies, Erwin and Castelli (2008) found that participation in physical activities did not contribute to the development of motor competence; similarly, Okely et al. (2001) found no significant relationship between non-organized physical activities and levels of motor competence. In addition to the need to resolve this inconsistency in findings, more research is needed on the development of motor competence in children aged 7–12 years (middle childhood) compared to, e.g., early childhood.

A less examined potential relationship is that between play (as a type of LTPA) and gross motor competence. Play is typically defined as locomotor activities that are primarily non-formal (i.e., simply done for their own sake) and give children excessive energy and joy (Roussou, 2004; Smith & Pellegrini, 2023). Children’s play is important from a public health perspective in terms of physical and mental health (Dodd et al., 2023), and it allows children to adapt to other children in various environments, which can have a positive impact on their motor development (Sutapa et al., 2021). Play is an essential and natural arena for learning various skills, and it might promote exercise (O’Connor & Stagnitti, 2011) as well as a transfer effect between physical education and spontaneous play (Pesce et al., 2016).

There is evidence for a link between level of play, physical activity intensity, and motor competence, as children can choose to be physically active or not in their play (Fisher et al., 2005), and more active play might benefit their motor competence. According to one study, having enough space for free play can improve motor competence, with one significant predictor being the playground size (True et al., 2017). Other studies have predicted that risky play—defined as thrilling forms of play that involve a risk of injury (Sandseter, 2009)—may improve children’s motor competence, as it allows children to explore affordances and test their skills (Brussoni et al., 2017). However, it is unclear how motor competence influences children’s physical activity during free play (Tsuda et al., 2020).

Another underexamined potential component of LTPA with respect to its relationship with gross motor competence is the potential for movement experiences occurring in the transportation children take to/from organized/non-organized activities and school. Specifically, active school transport (AST) can be defined as transportation from one general place to another general place by walking, biking, or other forms of non-motorized personal mobility (Faulkner et al., 2009). AST has the potential to be an important source of childhood physical activity (Larouche et al., 2024) and locomotor experiences. Additionally, children often allocate time to explore and play on their way to and from school, contributing to a potentially rich variety of motor behaviors that might have a positive impact on gross motor competence. Furthermore, spending time engaging in AST tends to increase the possibility of being physically active in other contexts (Stewart et al., 2017). Thus, children who do not engage in AST (or other physically active transport) and rely on bus rides or car rides might lose valuable time spent engaging in locomotor behaviors, which can have an impact on their motor development.

As with measuring physical activity in children, methodological issues have been raised concerning the predominant approaches to assessing children’s gross motor competence (Hulteen et al., 2023). Various authors have identified a focus on tasks with somewhat lower complexity (e.g., static tasks), detailed instructions, and highly specific scoring procedures, which are conducted in specific test facilities with isolated contextuality (Kennedy et al., 2013; Palmer et al., 2021). Concerns of ecological validity apply to this general approach, as the motor behavior - and corresponding motor competence level that children display emerge from the defining and interacting motor, perceptual, cognitive, and affective features inherent in the test design (Button et al., 2020; Handford et al., 1997). Another concern is related to the operationalization of task performance, where test batteries used to capture motor competence are often either product-oriented (e.g., quantitative outcomes) or process-oriented (e.g., qualitative characteristics). However, in real-world, dynamic environments, children experience multiple requirements inherent to gross motor skills, and incorporating such features requires a greater emphasis on multi-factorial integration in a dynamic context.

Adopting more representative task designs can allow children to apply and adjust different coordination patterns to a greater extent so as to effectively satisfy the interacting constraints involved in task exploration (Araujo et al., 2007). This, in turn, enables the assessment of the children’s capabilities to continuously adapt, modify, and regulate their gross movement patterns. Children with more experience and potentially better control of their gross movements in one domain might be capable of transferring these into other motor domains to their advantage, a possibility addressed by Bernstein (1967) theory of degrees of freedom (DF). The concept of freeing/freezing DF has been extensively studied as a key theoretical framework that describes how multiple independent components (bones, joints, muscles, etc.) can be combined in countless ways, all controlled by a single effector system (the central nervous system) (Guimarães et al., 2020). This concept captures the many different possibilities of movement solutions that may lead to the same outcome: since joint angles are abundant, there are more available joint movement combinations than necessary (Golenia et al., 2018; Latash et al., 2002), but one movement solution may be more inefficient than another (Gray, 2020). Children with better gross motor competence may be able to adjust DF to a greater extent and, therefore, have a larger motor repertoire with which to explore and handle more demanding challenges. Indeed, the conceptualization of freezing/freeing DF as a movement strategy has been systematically connected to various motor performance levels, in addition to being an integrated feature of the motor learning process (Gray, 2020; Guimarães et al., 2020; Palmer et al., 2021).

Building on this understanding, an alternative approach to integrating ecological perspectives in assessments of gross motor competence in children, as well as adopting a more representative task design, is the use of virtual reality (VR). This innovative technology can use real-world inputs to a person’s sensory neural circuits, thereby supporting their investigation of their actions and stimuli in response (Minderer et al., 2016). VR has been successfully used in the investigation of sports, rehabilitation, traffic safety, gaming, and injury prevention (Harris et al., 2020; Liu et al., 2020; Rimer et al., 2021; Sandseter et al., 2023; Schwebel et al., 2016; Theodoropoulos & Antoniou, 2022), and may contribute valuable information regarding complex movements, as these are difficult to study due to uncontrolled external influences (Levac et al., 2019).

**Figure 1.**
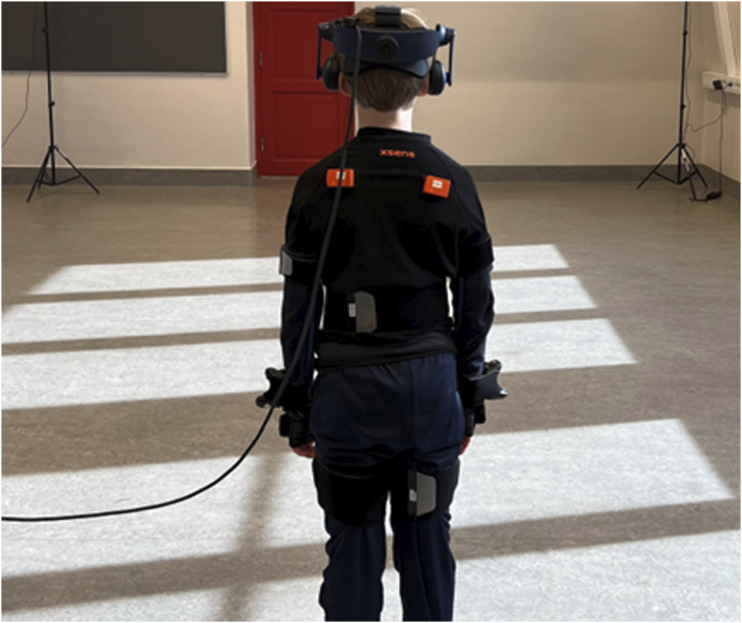
A child equipped with the VR and motion capture system, with Velcro bands.

In the current study, a virtual playground scenario (see Figure 1) was used to immerse children in a new and secure environment, wherein they could explore without specific instructions other than “try not to fall off”. To investigate the child’s motor behavior in the playground, whole-body inertial motion capture were applied, as these measures previously has been used to observe emerging motor behavior in the VR playground (Storli et al., 2024), revealing distinct movement variability patterns, including high variability in upper- and lower-body movements (indicative of children using large locomotion movements) or low variability in upper- and lower-body movements (indicative of careful movement patterns). The study also observed movement profiles combining high and low movement variability in the upper body and lower body (Storli et al., 2024).

Based on the presented considerations, the main aim of the current study was to investigate the association between parent-reported leisure-time physical activity levels and the children’s emergent motor behavior in a virtual reality playground scenario. It was hypothesized that children with higher reported frequencies of leisure-time physical activities would demonstrate more active exploration in the virtual reality playground. Further on, it was hypothesized that those more engaged in leisure-time physical activities would have more dynamic whole-body movement patterns with more freeing of degrees of freedom.

## Methods

### Participants

Part of a larger study called Virtual Risk Management (ViRMa) (Sandseter et al., 2023) the current study includes a sample of *n* = 247 children, mean age 8.79 years (SD .84), recruited from four primary schools in Norway. Participants were recruited through contact with the schools’ principals and then with guardians via an online platform. To avoid excluding any children from a particular setting, the four primary schools were from different parts of Norway and invite all children in the relevant age range at each school were invited to participate. Inclusion criteria were (1) between the ages of 7 and 10 years (grades 2, 3, and 4 in Norwegian schools), (2) informed consent obtained from parents to participate, and (3) sent obtained from the child to participate. The children in the current sample thus came from two urban schools (*n* = 177) and two rural schools (*n* = 70). The gender distribution in the sample was balanced, with 127 girls and 120 boys.

The parents/guardians of the participating children were invited to complete a questionnaire that included items on the child’s participation in leisure-time physical activities. The ViRMa project was approved (ref. no. 784782) by the Norwegian Agency for Shared Services in Education and Research (Sikt) and adheres to Sikt’s ethical guidelines in handling all project-related data and information. Informed consent from parents/guardians was obtained prior to data collection, and children gave their assent before starting the virtual reality tasks; both guardians and children were informed about their right to withdraw from study at any stage.

### Procedures

Data was collected over an 8-week period, from January to April 2023. The measurements were obtained during school time, with one child at a time escorted from the classroom to the test location at the child’s school (e.g., in a gymnasium or large classroom). Children’s body heights and foot lengths were measured as the rest of the equipment (VR headset and motion capture IMUs) was put on. For standardization purposes, all children participated without shoes and were asked to wear light clothing such as t-shirts or wool shirts. After the initial measurements and assembly of equipment, each child spent some minutes in an introduction scenario in VR to familiarize themselves with the circumstances; in this scenario, calibrations for the motion capture system were conducted. The children then individually performed the tasks. The same researcher escorted the child from the classroom, put on the equipment, and walked beside the child in order to remain nearby if anything should happen (e.g., falls, or if the child had questions or wanted to withdraw from the study). The children were able to communicate with the researcher at any time during the VR task if they felt that the task was too challenging.

### Apparatus

The VR equipment HTC VIVE Pro was installed at each participating school with an area of 6 × 5 m. A calibration of the VR software (Steam VR 2.0) using four base stations established the area within which the children moved during the VR tasks. VR was used to create the environment where the child should perform the play task in, as an attempt to give the participant an environment that seems familiar to everyday life. No physical elements were located within the calibrated area, so participants moved within this area and received all the information, sounds, and experiences through the VR system. The software captures data at 90 Hz. Each child was equipped with an HTC VIVE Pro headset and five VIVE 3.0 trackers, one on each foot and hand, and one in front of the stomach attached with Velcro bands. The trackers on the feet created transparent shoes in the VR environment so the child could see their feet. For gathering information about the children’s movements, seventeen sensors (IMUs) from the Xsens Awinda system, each 47 × 30 × 13 mm in size and weighing 16 g, were placed around the participants’ bodies using Velcro bands (Figure 1). These motion-caption sensors were strategically placed in the following locations: forehead, sternum and shoulders, hands, upper arms, lower legs, and upper legs.

The IMUs capture data at a rate of 60 Hz, offering 3D kinematic information on position, linear and angular acceleration, and velocity for 23 segments. Additionally, they provide 3D joint angles for 22 joints; these measurements are taken along three orthogonal axes in the global reference system and thus provide motor behavior data on the children as they explore the VR playground.

### The Virtual Reality Playground

**Figure 2.**
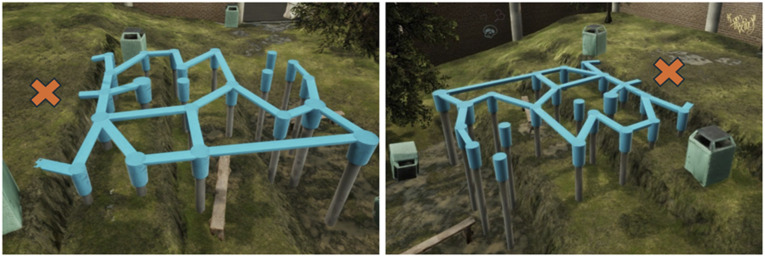
Overview of the virtual reality playground scenario. The red X marks the initial starting position for the child.

The playground simulated an urban city environment consisting of pillars and balance beams for the children to explore (see Figure 2). At the beginning of the scenario, children were provided with in-ear information through a pre-recorded female voice telling them that they were free to move, explore, and play as they like, but to try to avoid falling from the structures.

If the child fell off, the voice told the child that they had fallen off, and the software allowed the child to return to the starting point and to resume exploring freely. The scenario did not contain a fixed objective for the children to achieve; they could move around by balancing on the blue beams and/or jumping between the different pillars. The absence of fixed goals is theoretically justified by the notion of ecological validity, which emphasizes creating environments that closely mirror real-world settings to elicit more natural behaviors. By not imposing specific objectives, children are allowed to engage in self-directed exploration and play, which is more representative of emergent motor behavior.

The further they explored on the play stand, the greater ‘height’ the children reached, as the ground below them seemed further away; the height differences were zero cm in zone 1, 80 cm in zone 2, 145 cm in zone 3, and 235 cm in zone 4. The various heights were created to see if children’s movements differed in low and high heights. The width of the balancing beams was 20 cm. In order to create a realistic experience, the VR headset played sounds from passing cars, people, and birds, enhancing their immersive VR experience while also incorporating environmental factors that could potentially affect their movements. The children were able to see transparent shoes from the feet trackers in the task; the visual part of the foot in the VR environment was 20 cm long and 7 cm wide based upon average measure of children’s foot length aged 7–10-year-old. The time limit of the scenario was 3 minutes.

### Parent Questionnaire: Leisure-time Physical Activities

To gather background information about the children’s leisure-time physical activities, all parents/guardians were invited to answer a questionnaire. The survey was administered via an online platform using the survey software SurveyXact (Rambøll Management Consulting, Oslo, Norway). The questionnaire consisted of items in the form of “*How often does the child usually engage in these activities?*”; the survey respondents answered what was typical for the child’s activity during the last year, with answers on a scale of (1) Never, (2) 2–3 times a month or rarer, (3) 1 time a week, (4) 2–3 times a week, and (5) 4 times or more a week. The items asked about the child’s participation in organized sports, outdoor play with adult supervision, outdoor play *without* adult supervision, non-organized outdoor activities in nature, swimming, and several sports. In addition, the survey asked about the child’s transport experiences, such as walking/biking to school, shopping center, visits to friends/family, and playground. The items in the questionnaire were adapted from validated items in The Trøndelag Health Study (HUNT) - a longitudinal population health study in Norway that has been ongoing for four decades (Kurtze et al., 2007, 2008). For those children who had both parents complete the questionnaire, the answers were compared, and if these were not the same, an average of the two answers was calculated (this applied to 29 of the responses).

### Data Analysis

There seems to be a consensus in the literature that children 6 years and older are recommended to have at least 60 minutes of moderate-to-vigorous physical activity each day and a minimum of three high-intensity cardio-focused activities per week (Helsedirektoratet, 2023; Piercy et al., 2018). Given Norwegian and international recommendations, the current study classified low and high participation in an leisure-time physical activities based on the parent responses regarding their child’s engagement in the activity: if guardians reported never, 2–3 times a month or less, or 1 time a week, their children were coded as low engagement; if guardians reported 2–3 times a week or 4 times a week or more, their children were categorized as high engagement (see Table 1). This categorization was also used for overall engagement in leisure-time physical activities.

**Table 1.** Descriptive Statistics for Leisure-time Physical Activity Levels (*n* = 247).

Variable		1	2	3	4	5
Sport participation		20 (8.1%)	7 (2.8%)	74 (30.0%)	130 (52.6%)	16 (6.5%)
Number of sports	Mean: 3.0, SD: 1.8	-	-	-	-	-
Play outdoor	With supervision	11 (4.5%)	58 (23.5%)	81 (32.8%)	65 (26.3%)	32 (13.0%)
	Without supervision	6 (2.4%)	45 (18.2%)	55 (22.3%)	97 (39.3)	44 (17.8%)
Non-organized outdoor activities		16 (6.5%)	111 (44.9%)	83 (33.6%)	34 (13.8%)	3 (1.2 %)
Active transport	School	45 (18.2%)	13 (5.3%)	6 (2.4%)	25 (10.1%)	158 (64.0%)
	Sports	148 (59.9%)	30 (12.1%)	38 (15.4%)	24 (9.7%)	7 (2.8%)
	Visit	39 (15.8%)	49 (19.8%)	45 (18.2%)	71 (28.7%)	43 (17.4%)
	Playground	37 (15.0%)	59 (23.9%)	55 (22.3%)	59 (23.9%)	37 (15.0%)
	Shopping	134 (54.3%)	67 (27.1%)	37 (15.0%)	7 (2.8%)	2 (0.8%)
	Mean 13.6, SD: 4.5	-	-	-	-	-

Movement data files were reprocessed in the MTv Awinda software (Xsens, Enschede, Netherlands), to ensure that all data were transferred and retransmitted. Raw data were exported and further processed in Matlab R2022a (MathWorks Inc, Natick, MA, USA) using in-house algorithms. After an inspection of the frequency spectrum content of the raw data with the periodogram method, a low pass, zero phase, fourth order, Butterworth filter was applied; cutoff values for the filter varied between 5 and 15 Hz, depending on the specific frequency content of the analyzed movements. The data was further visually inspected to ensure that stationary beginnings and/or ends (children not moving) of raw signals were removed from further analysis. The following joint variables were studied for upper-body movement variability: shoulder abduction-adduction, elbow flexion-extension, head yaw, and pitch. In the analysis of lower-body movement variability, ankle dorsiflexion-extension, knee flexion-extension, and hip flexion-extension were examined. The distances between foot segments in mediolateral, anterior-posterior, and vertical directions were also included. Individual whole-body movement patterns were investigated through variability in freeing/freezing DF, as children’s movements were measured continuously for 3 min as they navigated the scenario. The standard deviation (SD) across the 3-min period was applied as a proxy for freeing/ freezing DF; it was computed for each individual joint across the entire movement time. The SD represents a common metric used to study variability in joint range of motion and other coordinative patterns (Gray, 2020; Kelso & Zanone, 2002; Rosengren et al., 2009; Wagner et al., 2012). The rationale for the movement analysis is explained in further detail in Storli et al. (2024).

### Statistical Analysis

Prior to the analysis, all variables were tested for normality using non-significant Kolmogorov-Smirnov tests and were inspected using histograms and Q–Q plots. The occurrence of missing values was low at < 1%. Little (1988) test of Missing Completely at Random (MCAR) indicated that data were missing completely at random (*χ2* = 387, *df* = 416, *p* = .833). Missing values were thus treated by replacing them with mean values. Descriptive statistics were further computed for all variables. Pearson’s correlations were used to explore the relationships between variables, and highly correlated variables were converted based on the mean value: this applied to the left and right sides of the shoulder, elbow, hip, and knee, given that the children moved in both mediolateral and anteroposterior directions around the playground. A principal component analysis (PCA) with Varimax rotation was used to examine the dimensional structures of the correlation matrices; the PCA was conducted on a dataset compromising six variables for the upper extremities and six for the lower extremities (see Table 2). The Kaiser-Meyer-Olkin measure of sampling adequacy and Bartlett’s test of sphericity were used to assess whether the correlation matrices were suitable. Potential differences in playground behavior between children with high and low levels of engagement in LTPA were examined using independent samples *t*-tests; Cohen’s *d* was applied as a measure of the size of the effect, whereby 0.2, 0.5, and 0.8 were considered to indicate small, moderate, and large effects, respectively (Lakens, 2013) as well as with chi square tests for categorical variables. All statistical calculations were performed in SPSS Predictive Analytics (IBM, Armonk, NY, United States) version 29.0, with α = 0.05 as the criterion for statistical significance.

**Table 2.** Descriptive Statistics for Whole-Body Movement Variability and Spatiotemporal Measures in Playground (*n* = 247).

Movement variability	Mean (SD)
Upper body	
Head – Yaw (deg°)	10.85 (4.17)
Head – Pitch (deg°)	9.11 (5.21)
Shoulder (abduction-adduction, deg.°)	8.27 (3.24)
Elbow (flexion-extension, deg.°)	19.92 (10.32)
Forearm height (%)	.04 (.03)
Trunk acceleration (m/s^3^)	.51 (.16)
Lower body	
Mediolateral feet distance (cm)	.114 (.027)
Anterior posterior feet distance (cm)	.110 (.026)
Vertical feet distance (cm)	.033 (.008)
Ankle (dorsiflexion-extension, deg.°)	9.03 (2.21)
Knee flexion-extension, deg.°)	14.84 (4.64)
Hip flexion-extension, deg.°)	13.54 (4.76)
Spatiotemporal playground measures	
Distance (m)	36.53 (12.48)
Velocity (m/s^2^)	.20 (.06)
Fall (yes/no)	Yes: 63 (25.5%), no: 187 (74.5%)
Visiting pillars (*n*)	2.66 (2.8)

## Results

Descriptive statistics appear in Tables 1 and 2 It emerges from the children’s demographics that 99.2% of the sample attended kindergarten for an average of 5 years. Most children spend all their first years in kindergarten as Norwegian children enter school and first grade by the age of 6.

### Sports Participation

**Figure 3.**
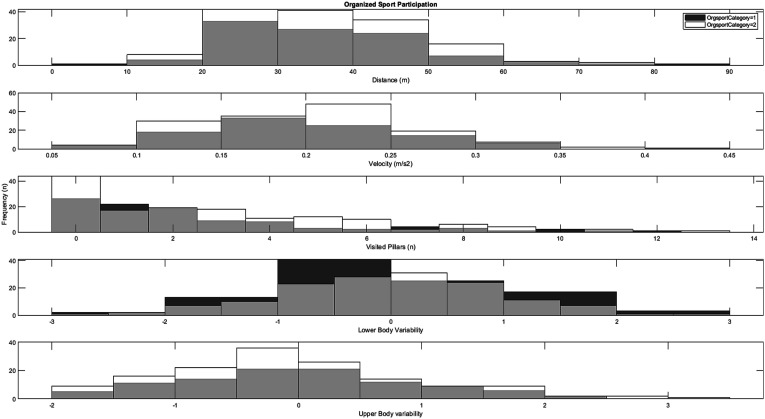
Histograms for the distribution of playground variables across low weekly participation rates (category 1) and high weekly participation rates (category 2) in organized sport.

As can be seen in Figure 3, independent samples *t*-tests indicated no significant differences between children with reported low (≤1 time a week) weekly participation in organized sports (*n* = 101) compared to children with reported high (≥2–3 times a week) weekly participation in organized sports (*n* = 146) on any of the measures measured in the virtual playground: distance (*t* = .07, *df* = 245, *p* = .94, *d* = .01), velocity (*t* = .04, *df* = 245, *p* = .97, *d* = .01), visited pillars (*t* = .83, *df* = 245, *p* = .40, *d* = .11), fall off balance beam (*χ*^*2*^ = 3.43, *df* = 1, *p* = .06), and movement variability (upper body: (*t* = .63, *df* = 245, *p* = .53, *d* = .08), lower body: (*t* = .03, *df* = 245, *p* = .97, *d* = .01)). Similarly, there were no significant correlations between any of the playground measures and the number of sports the children played (*r* ≤ .09, *p* ≥ .14).

### Engagement in Outdoor Play and Non-organized Activities

**Figure 4.**
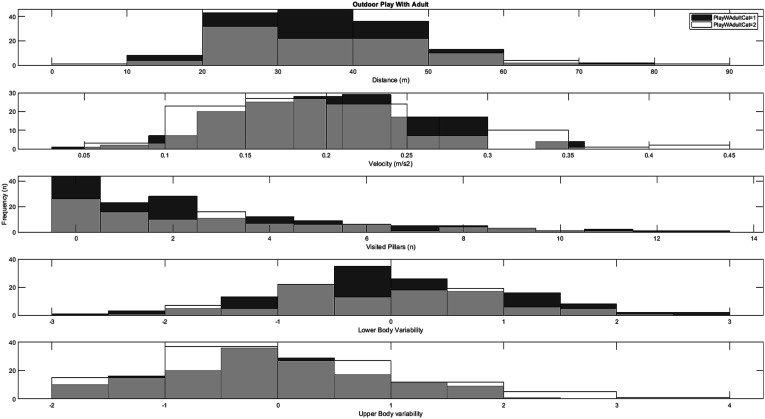
Histograms for the distribution of playground variables across low weekly participation rates (category 1) and high weekly participation rates (category 2) in outdoor play with adult supervision.

**Figure 5.**
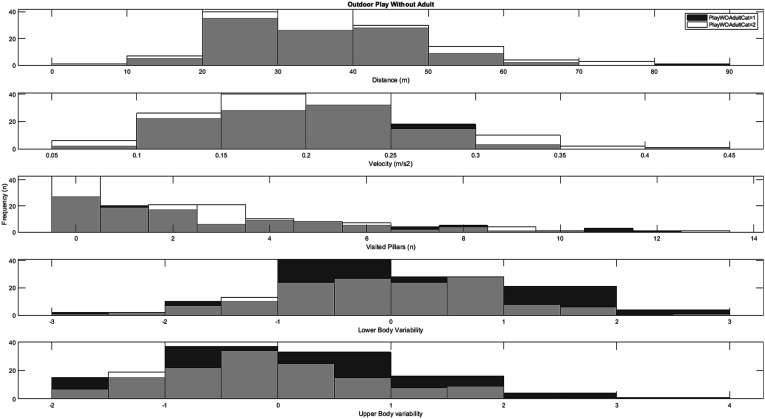
Histograms for the distribution of playground variables across low weekly participation rates (category 1) and high weekly participation rates (category 2) outdoor play without adult supervision.

The associations between reported levels of outdoor play and spatiotemporal/kinematic playground measures are depicted in Figures 4 and 5.

The analysis of children with reported high (≥2–3 times a week, *n* = 97) weekly engagement in outdoor play *with* adult supervision compared to children with reported low (≤1 times a week, *n* = 150) weekly engagement in outdoor play *with* adult supervision indicated no significant differences in motor behavior measured in the virtual playground: distance (*t* = 1.07, *df* = 245, *p* = .28, *d* = .14), velocity (*t* = .33, *df* = 245, *p* = .74, *d* = .04), visited pillars (t = .34, df = 245, *p* = .73, d = .05), fall off balance beam (*χ2* = 0.95, *df* = 1, *p* = .37), and movement variability (upper body: (*t* = .47, *df* = 245, *p* = .65, *d* = .06), lower body: (*t* = 1.07, *df* = 245, *p* = .29, *d* = .14)).

Independent samples *t*-tests for low (*n* = 106) versus high (*n* = 141) weekly engagement in outdoor play *without* adult supervision also indicated no significant differences: distance (*t* = .49, *df* = 245, *p* = .63, *d* = .06), velocity (*t* = .05, *df* = 245, *p* = .96, d = .01), visited pillars (t = 1.07, *df* = 245, *p* = .29, *d* = .14), fall off balance beam (*χ2* = 0.01, *df* = 1, *p* = .56), and movement variability (upper body: (*t* = 1.54, *df* = 245, *p* = .06, *d* = .20), lower body: (*t* = 1.41, *df* = 245, *p* = .08, *d* = .19)).

**Figure 6.**
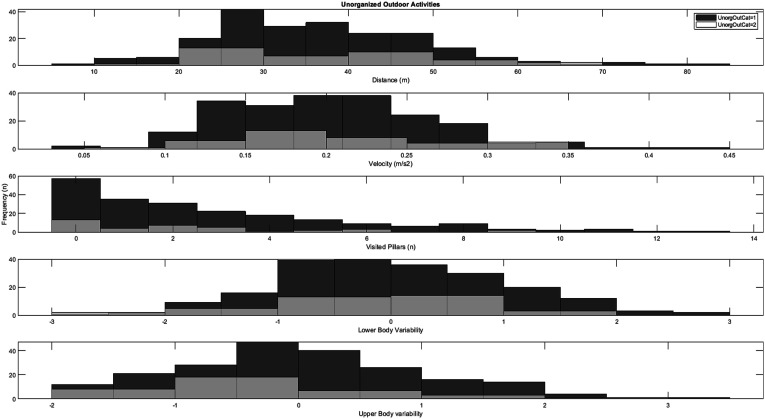
Histograms for the distribution of playground variables across low weekly participation rates (category 1) and high weekly participation rates (category 2) in unorganized outdoor activities.

Finally, independent samples *t*-tests for low (*n* = 106) versus high (*n* = 141) weekly engagement in non-organized outdoor activities (see Figure 6) indicated no significant differences for the variables distance (*t* = .37, *df* = 245, *p* = .71, *d* = .06), velocity (*t* = .01, *df* = 245, *p* = .99, d = .01), visited pillars (t = 1.06, *df* = 245, *p* = .33, *d* = .17), fall off balance beam (*χ2* = 0.99, *df* = 1, *p* = .41), and lower-body movement variability (*t* = 1.46, *df* = 245, *p* = .07, *d* = .26); however, a significant difference was found for upper-body movement variability (*t* = 2.02, *df* = 245, *p* = .02, *d* = .37).

### Active Transport

**Figure 7.**
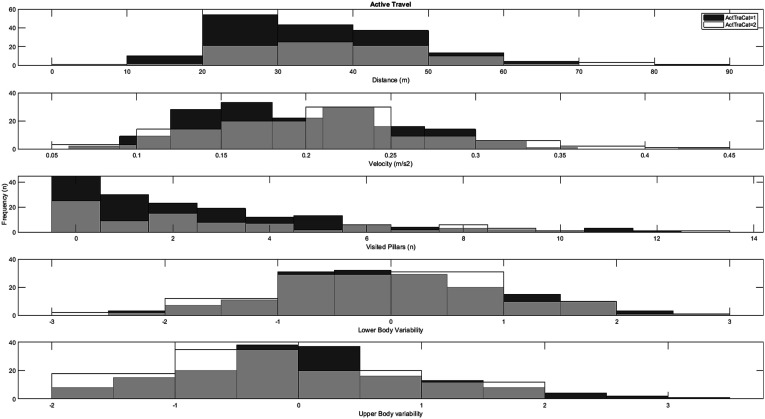
Histograms for the distribution of playground variables (distance, velocity, visited pillars and movement variability) across low weekly participation rates (category 1) and high weekly participation rates (category 2) in active travel.

As depicted in Figure 7, independent samples *t*-tests indicated no significant differences between children with reported low weekly engagement in active transport (*n* = 162) compared to children with reported high weekly engagement in active transport (*n* = 85) for the following measures in the virtual playground: distance (*t* = 1.67, *df* = 245, *p* = .09, *d* = .23), velocity (*t* = 1.48, *df* = 245, *p* = .14, *d* = .21), visited pillars (*t* = 1.11, *df* = 245, *p* = .29, *d* = .15), fall off balance beam (*χ*^*2*^ = .99, *df* = 1, *p* = .32), and lower-body movement variability (*t* = .59, *df* = 245, *p* = .55, *d* = .08). A significant between-group difference was found for upper-body movement variability (*t* = 1.98, *df* = 245, *p* = .03, *d* = .27).

### Overall Engagement in Leisure-time Physical Activities

**Figure 8.**
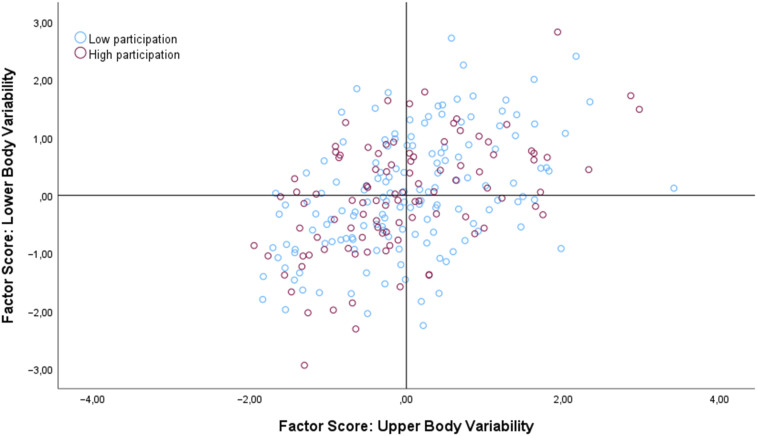
Scatterplot of the relation between upper and lower body movement variability across high and low overall weekly participation rates.

Statistical comparisons of children with overall low weekly engagement in LTPA (*n* = 147, 59.5%) versus children with overall high weekly engagement in LTPA (*n* = 100, 40.5%) indicated no significant difference for the spatiotemporal measures from the virtual playground—distance (*t* = 1.31, *df* = 245, *p* = .19, *d* = .17), velocity (*t* = 1.18, *df* = 245, *p* = .24, *d* = .15), visited pillars (*t* = 0.12, *df* = 245, *p* = .90, *d* = .01), and fall off balance beam (*χ2* = .02, *df* = 1, *p* = .50)-and no significant differences (see Figure 8) for lower-body or upper-body movement variability (*t* = .51, *df* = 245, *p* = .61, *d* = .07).

## Discussion

The principal aim of the current study was to examine the association between children’s leisure-time physical activities (LTPA), as reported by parents, and their actual motor behavior in a virtual reality playground environment. It was hypothesized that children with higher levels of LTPA in the categories of active transport, play, and sports participation would demonstrate different motor behavior in the virtual playground than children with lower levels of LTPA. Our results provided partial support for this hypothesis, in a significant difference with a small-to-moderate effect size in upper-body movement variability between high and low engagement in the LTPA categories of play and active transport. However, there were no significant differences between children with high and low engagement in sports in either movement variability (see Figure 8) or spatiotemporal measures observed in the virtual playground. The same results were also found for children’s overall high versus low weekly engagement in general leisure-time physical activities.

In the current study, no significant differences in motor behavior on the virtual reality playground emerged between children with low weekly sports participation compared to those with high weekly sports participation (see Figure 3). This might be explained by the overall sports participation rate for the children in the sample: less than 10% were reported as not participating in any sport (see Table 1). This is typical for Norwegian children at this age, as national-level statistics indicate that 93% play a sport during childhood (Fredheim, 2017). Consequently, the current sample might be too homogenous in terms of athletic background to display any systematic variation in virtual playground motor behavior. Furthermore, while participation in both organized and non-organized sports activities have previously been shown to give children rich opportunities to enhance their motor competence/coordination, any given sport requires certain specific motor skills for successful participation (Holfelder & Schott, 2014; Vallence et al., 2019); this means that sport activities are typically conducted within a limited range of task and environmental constraints with the purpose of facilitating the acquisition of a specific motor skill. These might not transfer to more free and open-ended tasks, such as the virtual playground in the current study. In the context of school physical education (PE) in Norway, an arena where children also potentially could sample experiences that enrich their motor development processes (Lorås, 2020), there is also a clear tendency in Norwegian PE for a teaching focus on more physical capabilities and highly specific sport skills that can be practiced in indoor gymnasiums (Aasland et al., 2020; Vist Hagen et al., 2022). These features of both sport and physical education for children thus seem to require further work to disentangle the importance and overall contribution to motor development in middle childhood.

In contrast to the results for organized sports participation, significantly greater development was observed in upper-body movement variability among children who engaged in more outdoor play and non-organized activities (see Figures 4 and 5), albeit with small-to-moderate effect sizes. These differences might be explained by the content of such activities, as they typically involve a more comprehensive range of movements in more accessible settings (unlike the somewhat controlled environments typical of organized sports.) Since play has been shown to offer children opportunities for free choice of movement (Kola-Olusanya, 2005; Storli & Hagen, 2010), it may more closely resemble the instruction-free playground task used in the current study. Play is typically considered one of the most critical arenas for children to freely express themselves, and this study provides partial evidence for an association between engagement in play and motor development linked to upper-body movement variability. From the perspective of the theory of degrees of freedom (DF) (Bernstein, 1967), play might offer children the opportunity to experiment with multiple movement strategies in various environments, which could potentially enhance their ability to coordinate and control their movements. Children between the ages of 6 and 10 typically have more diversity in their leisure-time physical activities compared to older children; this might lead children with higher rates of play to develop a more adaptable and flexible motor repertoire, compared to children with little engagement in play activities. Nevertheless, no significant differences were found for the spatiotemporal measures between high and low weekly engagement in outdoor play and non-organized activities. These results align with Okely and colleagues (2001), who also reported no significant relationship between non-organized activity and locomotor skills (such as running and vertical jumping) in a process-oriented assessment. The potential association, significance, and mechanisms of engagement in play vis-à-vis motor development in 7–10-year-old children thus require further study.

As with the results for non-organized activities, a significant difference emerged in the current study for upper-body movement variability between the high and low weekly engagement groups of active school transport with small-to-moderate effect sizes (see Figure 7). This indicates that children with higher levels of weekly active transport exhibited slightly more variability in their upper-body movements during the virtual reality playground task than those with lower levels of weekly active transport. From an ecological dynamic perspective (Davids et al., 2012), active transport might offer children rich opportunities to explore various movement dynamics (e.g., biking, running, walking, climbing) under various environmental constraints (e.g., shifting terrain, surfaces, traffic, weather conditions, seasons, etc.). This might require children to adapt their movements to navigate these changes safely, as well as potentially demand postural control and balance. Of course, it is not possible to conclude from the data in the current study that children’s experience with active transport might have aided their development of balance and postural control, which in turn could have impacted their spontaneous motor behavior in the virtual playground. Nevertheless, one might postulate that children demonstrating higher levels of movement variability in the virtual playground—with its high demands for stability/balance—do so because their movement experiences have allowed for a greater degree of freeing and exploring their DF in control of their balance and posture under a variety of task and environmental constraints (Srinivasan & Mathiassen, 2012; Winter, 1995).

Kindergartens have a substantial potential impact on the motor development of Norwegian children. Norwegian kindergartens typically provide rich opportunities for exploring movement in a variety of environments—indoor and outdoor—all year long (Moser & Martinsen, 2010); many kindergartens spend considerable amounts of time in nature, which has been shown to impact physical/active play (Lysklett, 2013). Children enter a developmental phase characterized by high sensitivity to the environment due to high brain plasticity (Ekerdt et al., 2020; Martín-García & Rico-González, 2023). Thus, children in the current sample possibly have rich experiences with play from their years in kindergarten. Furthermore, given that (1) sports participation and active transport typically first begin around the age when school starts in Norway (6 years), and (2) sports are typically played once a week for this age group, these leisure-time physical activities might not have been acting for long enough to have a high impact in our study—where the age of the children was 7–10 years old—and their effects might first emerge later in childhood (Barnett et al., 2016).

As depicted in Figure 8, the association between the overall level of leisure-time physical activities (LTPA) and movement variability indicates no significant difference between groups. This means that the variance in movement variability for children with high LTPA engagement was like the variance for children with low LTPA engagement in the current sample. This highlights the importance of considering not only non-linear dynamics in children’s gross motor behaviors across various task and environmental constraints but also the nonlinearities in motor development and associated processes in 7-to-10-year-old children. Previous research in this age group has produced conflicting results regarding the importance of LTPA for children’s development in middle childhood (Erwin & Castelli, 2008; Okely et al., 2001). It is thus a novel finding of this study that children’s experience with active play and transport might impact their emergent and self-selected movement variability when they freely explore a playground task in virtual reality.

An essential feature of investigating children’s motor behavior is incorporating the interacting individual, task, and environmental constraints. Considering these intertwined processes may contribute to a broader understanding of children’s perceptual-motor behavior in various environments (Davids et al., 2012; Golding et al., 2014). Virtual reality opens possibilities for such explorations by providing controlled environments familiar to children’s real-world experiences, and studies have indicated that it can be used with young children (Lorås et al., 2023; Moe, 2020; Storli et al., 2024).

### Limitations and Future Directions

Although virtual reality technology can provide valuable knowledge on children’s behavior in realistic environments, one must acknowledge the lack of tactile information in such an environment, which may limit children’s perception of the reality of the task. Nevertheless, in the conversations with the participating children after the VR task in the current study, only seven children answered that they did not experience the playground task as realistic. This may also relate to previous use of virtual reality technology, as children are primarily familiar with the technology used for games. Another limitation is the lack of accurate background information regarding the child’s participation in leisure-time physical activities. This requires parents/guardians to retrospectively adjust for the child’s participation, which can lead to both overestimation and underestimation compared to measurements that directly capture the time spent in LTPA.

Although the sample provided valuable insight into the research questions, a larger sample and more diverse sample would have allowed for more robust statistical analyses and increased certainty in the results. Future research should thus aim to include a more diverse sample in terms of leisure-time physical activities to validate and extend the findings. Moreover, future research should continue investigating children’s motor behavior using exploratory tasks, as these potentially provide valuable information about movement variability that can otherwise be challenging to detect with instruction-based assessments.

## Conclusion

The results of the current study indicate that 7-to-10-year-old children with higher weekly engagement in parent-reported play activities and active transport demonstrate significantly higher upper-body movement variability when they freely explore a virtual reality playground task. No other significant differences were found between high and low levels of leisure-time physical activity in terms of emergent motor behavior on the playground (whole-body movement variability and spatiotemporal measures). Thus, specific movement solutions on the virtual reality playground were not associated with any leisure-time physical activity. The observed small-to-medium effect sizes for upper-body movement variability warrant further investigation to explore the specific influence of leisure-time physical activity on children’s motor control and coordination during middle childhood. While previous studies have predominantly examined the intensity of physical activity in relation to motor development in this age group, this study suggests that self-generated low-intensity movement experiences-i.e., active transport and play can potentially be linked to motor system development, if the complexity and interaction of product and process-oriented assessments are jointly considered in, e.g., various virtual reality environments.

## Data Availability

The data that support the findings of this study are available from the corresponding author, LS, upon reasonable request.*
